# MicroRNA *Hsa-Let-7b* Regulates the Osteogenic Differentiation of Human Periodontal Ligament Stem Cells by Targeting CTHRC1

**DOI:** 10.1155/2021/5791181

**Published:** 2021-12-14

**Authors:** Lin Fu, Na Li, Yu Ye, Xiaying Ye, Tong Xiao, Xiao Wu, Yanxia Ma, Jinhua Yu

**Affiliations:** ^1^Key Laboratory of Oral Diseases of Jiangsu Province and Stomatological Institute of Nanjing Medical University, 136 Hanzhong Road, Nanjing, 210029 Jiangsu, China; ^2^Endodontic Department, School of Stomatology, Nanjing Medical University, 136 Hanzhong Road, Nanjing, Jiangsu 210029, China

## Abstract

*Let-7* miRNA family has been proved as a key regulator of mesenchymal stem cells' (MSCs') biological features. However, whether *let-7b* could affect the differentiation or proliferation of periodontal ligament stem cells (PDLSCs) is still unknown. Here, we found that the expression of *hsa-let-7b* was visibly downregulated after mineralization induction of PDLSCs. After transfected with *hsa-let-7b* mimics or inhibitor reagent, the proliferation ability of PDLSCs was detected by cell counting kit-8 (CCK-8), flow cytometry, and 5-ethynyl-2-deoxyuridine (EdU) assay. On the other hand, the osteogenic differentiation capacity was detected by alkaline phosphatase (ALP) staining and activity, alizarin red staining, Western blot, and quantitative real-time reverse-transcription polymerase chain reaction (qRT-PCR). We verified that *hsa-let-7b* did not significantly impact the proliferation ability of PDLSCs, but it could curb the osteogenic differentiation of PDLSCs. Besides, we predicted CTHRC1 acts as the downstream gene of *hsa-let-7b* to affect this process. Moreover, the combination of CTHRC1 and *hsa-let-7b* was verified by dual luciferase reporter assay. Our results demonstrated that the osteogenic differentiation of PDLSCs was enhanced after inhibiting *hsa-let-7b*, while was weakened after cotransfection with Si-CTHRC1. Collectively, *hsa-let-7b* can repress the osteogenic differentiation of PDLSCs by regulating CTHRC1.

## 1. Introduction

Periodontal disease is a chronic inflammatory disease, which usually leads to periodontal tissue destruction (including gingival bleeding, periodontal pocket formation and inflammation, attachment loss, and alveolar bone absorption) [1]. If not treated in time, it will lead to tooth loosening or even extraction [1, 2]. The ultimate aim of periodontal therapy is to promote the regeneration of periodontal tissue [3]. Traditional treatment can effectively control periodontal inflammation, but effective methods for periodontal tissue regeneration are still lacking up to now [4].

Recently, mesenchymal stem cell-based therapy has exhibited a good prospect in improving the healing, repair, and regeneration of periodontal tissue. PDLSCs are one of the most widely studied MSCs, which are regarded as the most suitable cells for periodontal tissue repair and regeneration [2]. Studies have shown that PDLSCs have multidirectional differentiation potential and strong self-renewal ability. These cells can differentiate into bone, adipose, and cartilage tissue [5]. Ding et al. [6] and Mrozik et al. [7] found that repair or regeneration can be observed after transplanting autologous or allogeneic PDLSCs into periodontal lesions. In addition, some studies have found that autologous PDLSC transplantation can increase the bone height of intraosseous defects [8]. Therefore, understanding the molecular mechanism of PDLSC osteogenesis is advantageous to develop the regeneration treatment of periodontal and bone disease [5].

MicroRNAs (miRNAs) are small noncoding functional RNA molecules with a length of about 22 nucleotides. They directly bind to the 3′-untranslated region (3′-UTR) of messenger RNA (mRNA) in a sequence-specific manner, blocking translation or leading to mRNA degradation, thus playing an important role in gene expression regulation [9]. Many biological functions are highly dependent on miRNAs, including cell proliferation, differentiation, signaling pathways, apoptosis, and metabolism [10]. There is growing evidence which suggests that miRNA is in connection with the regulation of osteogenic differentiation of MSCs by targeting related genes and signaling pathways [11]. *Let-7* family is one of the earliest miRNAs which has been proved to be a key regulator of cell differentiation [12]. Besides, its sequence and function are highly conserved among different species [13]. *Let-7* participates in the regulation of stem cell activity, including downregulating some target genes to maintain stem cell stemness or directly differentiate into lineage cells [13]. Moreover, previous study has proven that *hsa-let-7b* can repress the osteogenic differentiation of SCAPs by targeting MMP1 [14]. However, the effect of *let-7b* on the directional differentiation of PDLSCs remains unclear.

Collagen triple helix repeat containing 1 (CTHRC1) gene is located on human chromosome 8q22.3, encoding a 28 kDa secretory protein [15]. It is highly conserved in chordate animals, while there is no homolog found in lower species such as flies and worms. It was initially found in balloon injured rat arteries and normal rat arteries and related to atherosclerosis [15, 16]. Studies have demonstrated that CTHRC1 is related to many physiological and pathological processes, such as bone formation, developmental morphogenesis, inflammatory arthritis, and cancer progression [15–17]. CTHRC1 transgenic mice showed high bone mass by increasing osteoblast bone formation, while CTHRC1-deficient mice showed low bone mass due to decreased osteoblast bone formation [18]. In addition, CTHRC1 was upregulated in the process of mucosal tissue repair. As a consequence, CTHRC1 may be associated with bone formation [19]. However, up to now, there are few studies on whether and how CTHRC1 affects the osteogenic differentiation PDLSCs.

Since miRNA *hsa-let-7b* plays a significant role in the committed differentiation of stem cells, and CTHRC1 is forecasted to be the target gene of *hsa-let-7b* by bioinformatics analysis, we hypothesized that the *let-7b* may clarify a certain key mechanism for the directed differentiation of stem cells by regulating CTHRC1. In this research, we mainly examined whether and how *hsa-let-7b* affects the osteogenic differentiation of PDLSCs.

## 2. Materials and Methods

### 2.1. Separation and Culture of Cell

Healthy premolars of donors (12-16 year of age) who need to be extracted due to orthodontic treatment were collected from the Oral and Maxillofacial Surgery Department of Jiangsu Provincial Stomatological Hospital. Informed consent was obtained from the patient. Periodontal ligaments were separated from the middle third of root surfaces and digested with type I collagen (Sigma-Aldrich, USA) and dispase (Gibco, Life Technologies) for 20 minutes, then incubated in a complete medium including alpha minimum essential medium (*α*-MEM, Gibco, Life Technologies), 10% fetal bovine serum (FBS, Gibco, Life Technologies), and 1% penicillin/streptomycin in a humidified 5% CO_2_ atmosphere at 37°C. When the cell density reached 70-80%, they were passed to a new culture dish. PDLSCs between passages 3 and 5 were used in the research.

### 2.2. Cell Identification

#### 2.2.1. Flow Cytometry (FCM)

PDLSCs were collected and incubated with different antibodies (CD29-APC, CD90-FITC, CD73-PE, CD105- PerCP-Cy5.5, CD34-PE, and CD45-PE) at 4°C, in dark for 1 hour. After that, samples were analyzed by FACSCalibur (BD Biosciences, CA, USA).

#### 2.2.2. Multiple Differentiation

*(1) Osteogenic Differentiation*. PDLSCs were induced with osteogenic differentiation medium for 14 days. Then, the alizarin red S solution (pH 4.2, Sigma-Aldrich, USA) was used to stain. Mineralized nodules were observed and captured using an inverted microscope (Leica, Germany).

*(2) Adipogenic Differentiation*. To induce adipogenic differentiation, PDLSCs were fed with the adipogenic medium (Cyagen Biosciences Inc., USA) for 21 days. Medium 1 and medium 2 were alternated until the adipocytes appeared. When lipid droplets were clearly observed, the cells were fixed with 4% paraformaldehyde (PFA) for 30 minutes and stained with Oil Red O.

*(3) Chondrogenic Differentiation*. For the chondrogenic differentiation of PDLSCs, a Chondrogenic Differentiation Kit (Cyagen Biosciences Inc., USA) was applied, and the medium was refreshed every 3-4 days. Cartilage nodules were observed by staining with the Alcian blue.

### 2.3. Cell Transfection

*Hsa-let-7b* mimics (mimic, 50 nM), mimics control (mimic NC, 50 nM), *hsa-let-7b* inhibitor (inhibitor, 100 nM), inhibitor control (inhibitor NC, 100 nM), CTHRC1 small interfering RNAs (Si-CTHRC1, 100 nM), and control groups (Si-NC, 100 nM) were constructed by Ribobio Corporation. PDLSCs were transfected with riboFECT™ CP kit (Ribobio, Guangzhou, China) at 30–50% confluence.

### 2.4. Cell Proliferation Assay

#### 2.4.1. Cell Counting Kit-8 (CCK-8)

Transfected cells were reseeded into 96-well plate (2 × 10^3^ cells/well). Following manufacturer's instruction, PDLSCs were treated with CCK-8 regents (CCK-8; Dojindo, Japan) at 37°C for 2 hours on days 0, 1, 3, 5, and 7. At last, a microplate reader (Bio-Tek, Winooski, VT) was used to detect the OD values at 450 nm.

#### 2.4.2. 5-Ethynyl-2-Deoxyuridine (EdU) Assay

The proliferation capacity was measured using the Cell-Light™ EdU Apollo®567 In Vitro Imaging Kit (Ribo Biotechnology, China) according to manufacturer's instructions. After transfected cells reseeding in 12-well plate, cells were incubated with 100 *μ*M EdU labeling solution at 37°C for 4 hours. Following that, cells were fixed with 4% PFA. Next, cells were hatched with glycine and washed with PBS. Cells were then permeabilized with 0.5% Triton X-100 and treated with a 1× Apollo® reaction mixture. Hoechst 33342 was used for staining nuclei. After washing with PBS, cells were observed and captured by a fluorescence microscope (Leica, Germany). The cell proliferation rate was quantified and evaluated by ImageJ software.

### 2.5. Alkaline Phosphatase (ALP) Staining and Activity

Transfected cells were cultured with osteogenic-induced medium for 7 days. Then, PDLSCs were fixed with 4% PFA and washed with PBS twice. ALP staining was carried out using the NBT/BCIP staining kit (Beyotime, China), and images were captured by an inverted microscope (Leica, Germany). According to manufacturer's protocol, ALP activity was analyzed by ALP activity assay kit (Jiancheng, China).

### 2.6. Alizarin Red S (ARS) Staining and Quantitative Analysis

After the transfected cells culturing in mineralization induction for 14 days, cells were fixed with 4% PFA and stained with the alizarin red S solution for 15 minutes. A microscope was applied to picture and observe *t* mineralized nodules. Besides that, 10% CPC (Sigma-Aldrich) was used for quantitatively evaluating the mineralized nodules and examined at 560 nm.

### 2.7. Immunofluorescence (IF) Staining

Transfected PDLSCs were fixed with 4% PAF, infiltrated with Triton X-100 solution (Beyotime, China) for 12 minutes, and blocked with goat serum. Then, cells were washed by PBS twice and incubated with STRO-1, CTHRC1, RUNX2, and ALP primary antibodies (1 : 100) overnight. The next day, they were incubated in a mixture of secondary antibody with fluorochrome for 1.5 hours in the dark. Then, nuclei were stained with DAPI (Beyotime, China). Finally, the result was observed under the fluorescence microscope (Leica, Germany).

### 2.8. Western Blot

PDLSCs transfected for 3 days were washed by PBS twice. Then, total protein was harvested using RIPA buffer (Beyotime, China). A BCA kit (Beyotime Biotechnology, China) was put in to determine the concentration of different samples. Samples were loaded onto 10% SDS-PAGE and transferred onto PVDF membranes (Millipore, USA). Membranes were blocked with 5% nonfat milk and then incubated with the following primary antibodies overnight at 4°C: ALP (Abcam), RUNX2 (Abcam), OSX (Abcam), CTHRC1 (Proteintech), and GAPDH (Proteintech). After three washes with TBST, membranes were detected with appropriate secondary antibodies for 1 hour. Finally, the protein bands were visualized with Western Blotting Imaging System (GE Healthcare, USA). ImageJ software was used to quantify the results.

### 2.9. Quantitative Real-Time Reverse-Transcription Polymerase Chain Reaction (qRT-PCR)

Total RNA was collected by TRIzol reagent (Invitrogen, USA). Isolated RNA was reverse-transcribed to cDNA by PrimeScript RT Master Mix kit (Vazyme, China) thenceforth. Following from that, qRT-PCR was conducted with ChamQTM SYBR Green quantitative PCR Master Mix (Vazyme, China). *GAPDH* and *U6* were used as the reference gene. Relative gene expression was calculated by 2^–ΔΔCT^ method as previously reported [20]. The sequences of primes were depicted in Table 1.

### 2.10. Dual-Luciferase Reporter Assay

In 24-well plates, 293 T cells were cultured to approximately 80% confluence and then cotransfected with either wild type (WT) or mutant (MUT) luciferase reporter vector (800 ng) (GeneChem, China), Renilla Luciferase (5 ng) reporter vector, and 100 nM *hsa-let-7b* mimics or normal control. 48 hours later, the luciferase activity was examined by Luciferase Reporter Assay System (Promega, USA).

### 2.11. Statistical Analysis

All experiments were repeated in triplicate and expressed as mean ± standard deviation. Differences were compared by Student's *t*-test and one-way analysis of variance (ANOVA) in SPSS software and GraphPad Prism 5. *P* values < 0.05 were considered statistically significant.

## 3. Results

### 3.1. Cells Culture and Identification

**Figure 1:**
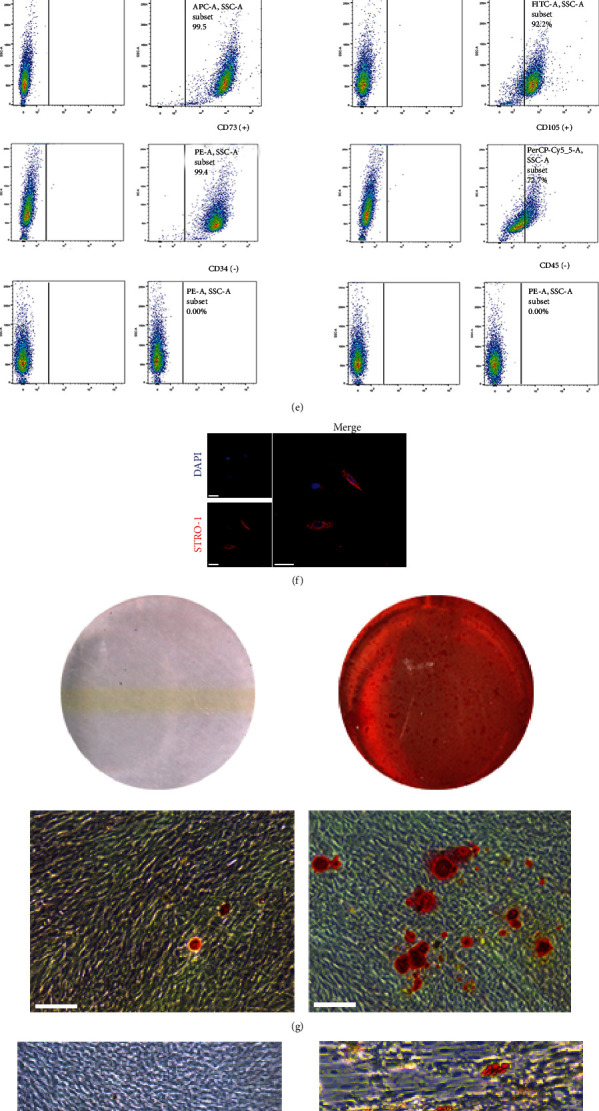
Identification of PDLSCs. (a) Imaging examination of immature permanent teeth. (b) Scraping the middle third of the root of the tooth. (c) The morphology of primary PDLSCs (scale bar = 100 *μ*m). (d) Morphology of the third-generation PDLSCs (scale bar = 100 *μ*m). (e) Flow cytometry assay showed that PDLSCs were positive for CD29, CD73, CD90, and CD105, but negative for CD34 and CD45. (f) Immunofluorescence assay revealed that PDLSCs were positive for STRO-1 (scale bar = 50 *μ*m). (g, h) PDLSCs had the potential to differentiate into osteoblasts and adipocytes (scale bar = 100 *μ*m). On the left is the control group which was induced by complete medium. (i) PDLSCs had the potential to differentiate into chondrocytes (scale bar = 200 *μ*m).

PDLSCs were obtained from the middle third of root surfaces of premolars successfully (Figures 1(a) and 1(b)). They exhibited typical spindle-like morphology observed under the microscope (Figures 1(c) and 1(d)). Flow cytometry assay-identified PDLSCs were highly positive for CD29, CD73, CD90, and CD105, but negative for CD34 and CD45 (Figure 1(e)). In addition, the following experiments (ARS staining, Oil red O staining, and Alcian blue staining) were performed in order to prove the multiple differentiation ability of PDLSCs. Results confirmed that PDLSCs could differentiate into osteoblasts, adipocytes, and chondrocyte (Figures 1(f)–1(h)).

### 3.2. Cell Transfection and Influence of *Hsa-Let-7b* on the Proliferation of PDLSCs

**Figure 2:**
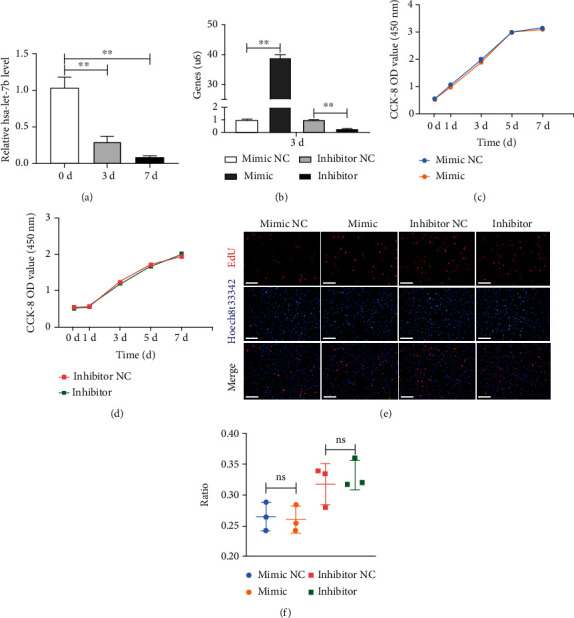
Transfection efficiency and the effect of *hsa-let-7b* on the proliferation of PDLSCs. (a) The expression of *hsa-let-7b* during the osteogenic differentiation of PDLSCs for 0, 3, and 7, days, respectively. ^∗∗^*P* < 0.01. (b) Transfection efficacy of *hsa-let-7b* was measured at day 3 by qRT-PCR. (c) CCK-8 analysis showed that there was no significant difference between mimic and mimic NC at 0, 1, 3, 5, and 7, respectively. *P* > 0.05. (d) CCK-8 analysis showed that there was no significant difference between inhibitor and inhibitor NC at 0, 1, 3, 5, and 7, respectively. *P* > 0.05. (e, f) Cell proliferation of PDLSCs was detected by EdU staining and assay. *P* > 0.05. EdU: 5-ethynyl-2-deoxyuridine; mimic: *hsa-let-7b* mimics; mimic NC: mimics control; inhibitor: *hsa-let-7b* inhibitor; inhibitor NC: inhibitor control.

To investigate the expression of *hsa-let-7b* in PDLSCs, cells were cultured with osteogenic-induced medium for 0, 3, and 7 days separately, and then, qRT-PCR was carried out. The results indicated that the expression was clearly downregulated especially on the third day (*P* < 0.01, Figure 2(a)). Besides, in order to detect whether *hsa-let-7b* impact the proliferation of PDLSCs, *hsa-let-7b* mimics and inhibitor were transfected into cells, and relevant transfection efficacies were measured (*P* < 0.01, Figure 2(b)).

CCK-8 and EdU assay were conducted to verify the influence of *hsa-let-7b* on proliferation of PDLSCs. CCK-8 results suggested that there was no visible discrepancy between group mimic NC and mimic (*P* > 0.05, Figure 2(c)), while group inhibitor NC also presented no dramatic difference compared with group inhibitor (*P* > 0.05, Figure 2(d)). Furthermore, EdU assay revealed similar results (*P* > 0.05, Figures 2(e) and 2(f)).

### 3.3. *Hsa-Let-7b* Affected the Osteogenic Differentiation of PDLSCs

**Figure 3:**
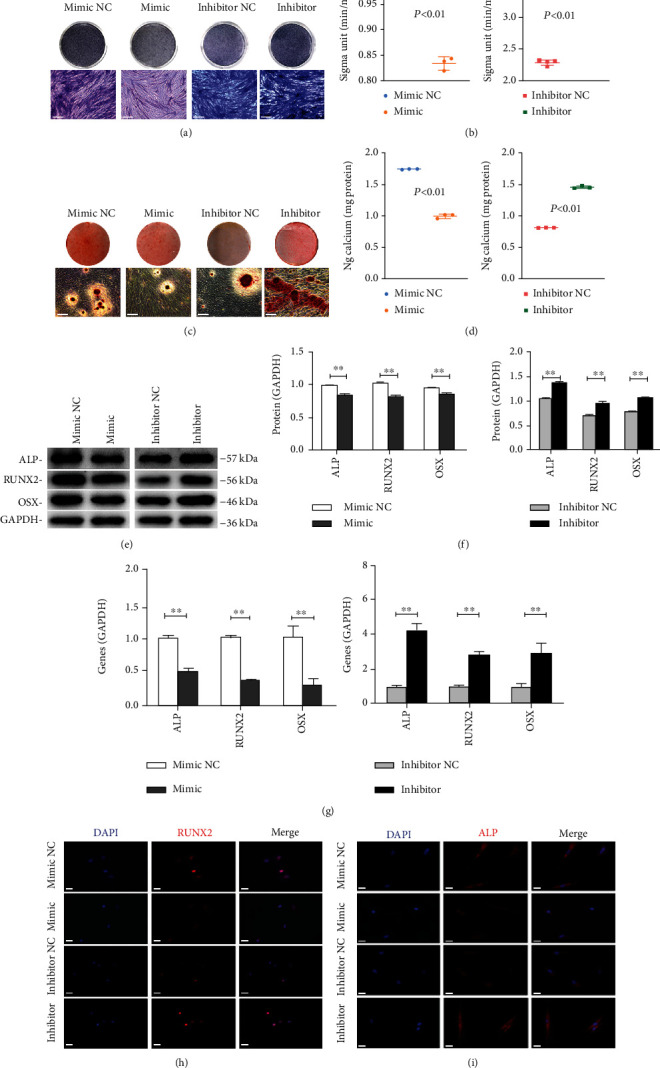
*Hsa-let-7b* inhibited the osteogenic differentiation of PDLSCs. (a, b) Results of ALP staining and ALP activity assay after 7 days of osteogenesis (scale bar = 100 *μ*m). ^∗∗^*P* < 0.01. (c, d) ARS staining and CPC assay at day 14 after osteogenic induction (scale bar = 100 *μ*m). ^∗∗^*P* < 0.01. (e) The expression of osteogenesis-related proteins (ALP, RUNX2, and OSX) after osteogenic induction 3 days of transfected PDLSCs. GAPDH served as the reference protein. (f) Relative grayscale analyses of (e). ^∗∗^*P* < 0.01. (g) The expression of osteogenesis-related genes (*ALP*, *RUNX2*, and *OSX*) in group mimic NC, mimic, inhibitor NC, and inhibitor at day 3, respectively. ^∗∗^*P* < 0.01. (h, i) The immunofluorescence staining of RUNX2 and ALP in transfected PDLSCs and matched controls (scale bar = 50 *μ*m).

To ulteriorly detect the functional roles of *hsa-let-7b* on the osteogenic differentiation of PDLSCs, cells were transfected with mimic NC, mimic, inhibitor NC, and inhibitor. After inducing osteogenesis for 7 days, ALP staining revealed that the staining areas were obviously decreased in group mimic as compared with that in group mimic NC, while group inhibitor showed completely opposite results (Figure 3(a)). ALP activity assay revealed the same results (*P* < 0.01, Figure 3(b)). After 14 days of osteogenesis, a similar trend in ARS staining was found (Figure 3(c)), as well as CPC analysis (*P* < 0.01, Figure 3(d)). Besides, protein and mRNA levels of ALP/*ALP*, RUNX2/*RUNX2*, and OSX/*OSX* were examined by Western blot and qRT-PCR. Results of the former confirmed that the protein expression of ALP, RUNX2, and OSX was cut down in mimic group, whereas it was upregulated when *hsa-let-7b* was suppressed (*P* < 0.01, Figures 3(e) and 3(f)). Similarly, results of latter also descended in mimic group, and the inhibitor group performed the contrary consequence (*P* < 0.01, Figure 3(g)). Moreover, the results of immunofluorescence verified that expression of osteogenic differentiation protein (RUNX2 and ALP) was the same as above (Figures 3(h) and 3(i)). To sum up, *hsa-let-7b* can obviously restrain the osteogenic differentiation of PDLSCs.

### 3.4. Bioinformatic Analysis on the Target Gene of *Hsa-Let-7b*

**Figure 4:**
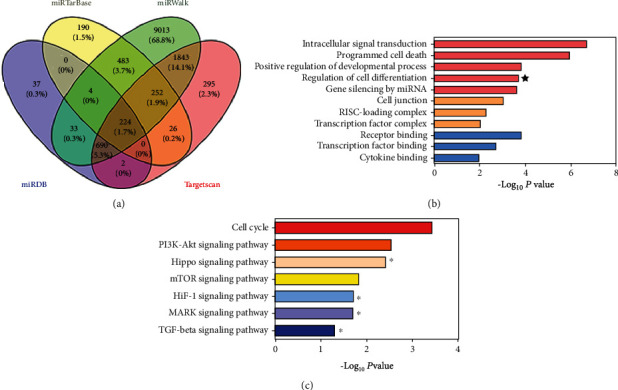
Bioinformatic analysis on the target gene of *hsa-let-7b*. (a) Venn diagram showed the number of *hsa-let-7b* target genes predicted by miRDB, miRTarBase, miRWalk, and TargetScan algorithms. (b) GO annotation suggested that these target genes are related to various biological processes. GO: domains directly related with reproduction. (c) KEGG pathway analysis indicated that these target genes are involved in kinds of cellular pathways.

Potential target genes (13,093) of *hsa-let-7b* were predicted using miRNA databases (miRTarBase, miRWalk, miRDB, and TargetScan algorithms) (Figure 4(a)). Among them, there were 224 common target genes. Then, GO annotation suggested that these target genes are concerned with various biological processes, such as cell differentiation (Figure 4(b)). Furthermore, KEGG pathway analysis indicated these target genes are relevant to kinds of cellular pathways, for example, TGF-*β* signaling pathway (Figure 4(c)). Coincidentally, CTHRC1 was a common target gene of *hsa-let-7b* in the 4 databases' prediction.

### 3.5. *Hsa-Let-7b* Directly Targets CTHRC1

**Figure 5:**
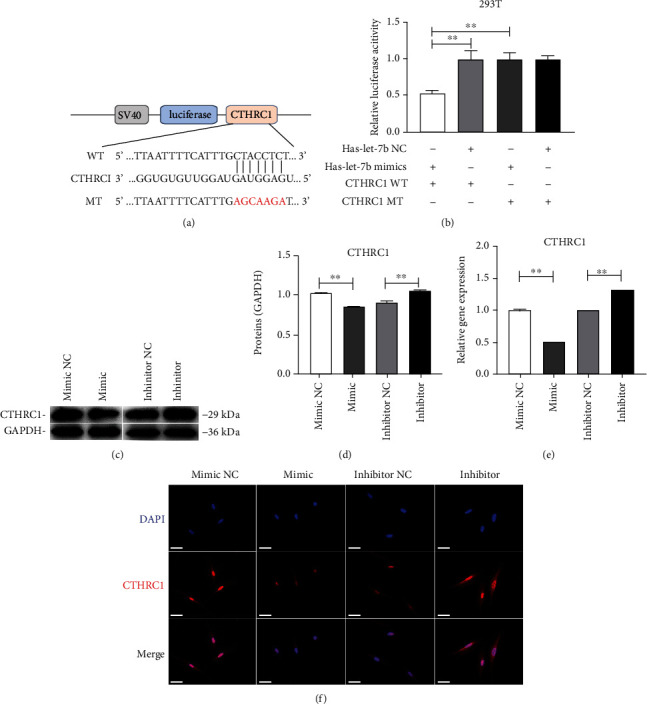
CTHRC1 is a direct target of *hsa-let-7b*. (a) The wild-type CTHRC1-3′-UTR (WT) and mutant CTHRC1-3′-UTR reporter plasmid (MUT) were constructed. (b) Luciferase reporter assays in 293 T illustrated that *hsa-let-7b* could bind with CTHRC1. (c–e) Western blot and qRT-PCR analysis were performed to detect the protein and mRNA levels of CTHRC1 in group mimic NC, mimic, inhibitor NC, and inhibitor at day 3, respectively. ^∗∗^*P* < 0.01. (f) The immunofluorescence staining of CTHRC1 in transfected PDLSCs and matched controls (scale bar = 50 *μ*m).

In order to verify whether *hsa-let-7b* directly regulates CTHRC1, luciferase reporter vector of the CTHRC1 3′UTR sequence containing the binding sites of *hsa-let-7b* was constructed (Figure 5(a)). The wild-type/mutant plasmid and *hsa-let-7b* mimic/mimic NC were cotransfected into 293 T cells, respectively, and then, the activity was detected. Luciferase activity was dramatically downregulated in group CTHRC1-WT+ *hsa-let-7b* mimic, while it was not observed in group MUT (*P* < 0.01, Figure 4(b)). Then, Western blot analysis and qRT-PCR were used to identify how *hsa-let-7b* regulates CTHRC1. Results showed that the expression of CTHRC1 decreased in mimic group, but increased in inhibitor group (*P* < 0.01, Figures 5(c)–5(e)). In short, CTHRC1 was regulated by *hsa-let-7b*.

### 3.6. CTHRC1 Promoted the Osteogenic Differentiation of PDLSCs

**Figure 6:**
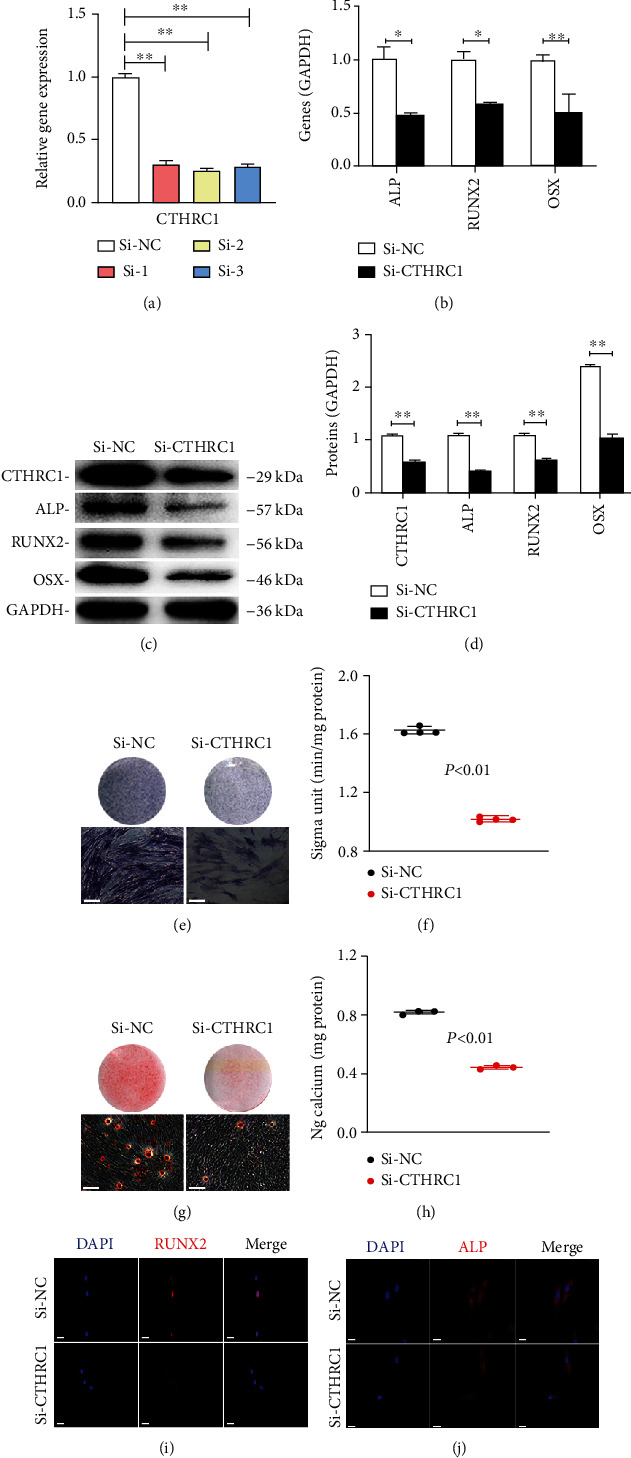
CTHRC1 enhanced the osteogenic differentiation capacity of PDLSCs. (a) The result of qRT-PCR showed that transfection effect of Si-2 was better. ^∗∗^*P* < 0.01. (b) QRT-PCR showed the expression of osteogenesis-related genes (*ALP*, *RUNX2*, and *OSX*) downregulated. ^∗∗^*P* < 0.01. (c) Western blot analysis of osteogenic markers in PDLSCs transfected with Si-CTHRC1. (d) Relative grayscale analyses of (c). ^∗^*P* < 0.05, ^∗∗^*P* < 0.01. (e, f) 7 days after mineralization, ALP staining and ALP activity analysis were performed (scale bar = 100 *μ*m). ^∗∗^*P* < 0.01. (g, h) Results of ARS staining and CPC assay at day 14 (scale bar = 100 *μ*m). ^∗∗^*P* < 0.01. (i, j) Immunofluorescence assay revealed that the protein expression of RUNX2 and ALP was downregulated in Si-CTHRC1 group as compared with controls (scale bar = 50 *μ*m).

In order to explore the effect of CTHRC1 on the osteogenic differentiation of PDLSCs, we constructed three siRNAs for CTHRC1. QRT-PCR analysis showed that after transfection with Si-CTHRC1, the expression level of CTHRC1 decreased significantly, and the inhibitory effect of Si-2 was better (*P* < 0.01, Figure 6(a)). Therefore, we chose Si-2 for the following experiment. Western blot analysis and qRT-PCR revealed the expression of ALP/*ALP*, RUNX2/*RUNX2*, and OSX/*OSX* was downregulated (*P* < 0.01, Figures 6(b)–6(d)). After 7 days of osteogenic induction, the staining and activity of ALP showed that Si-CTHRC1 group was clearly lower than Si-NC group (*P* < 0.01, Figures 6(e) and 6(f)). Alizarin red staining and relevant CPC quantitative analysis showed similar results (*P* < 0.01, Figures 6(g) and 6(h)). Besides, the result of immunofluorescence proved osteogenic-associated protein expression (RUNX2 and ALP) was identical as above (Figures 6(i) and 6(j)). In conclusion, CTHRC1 can enhance the osteogenic differentiation of PDLSCs.

### 3.7. CTHRC1 Partially Blocks the Impacts of *Hsa-Let-7b* on PDLSCs

**Figure 7:**
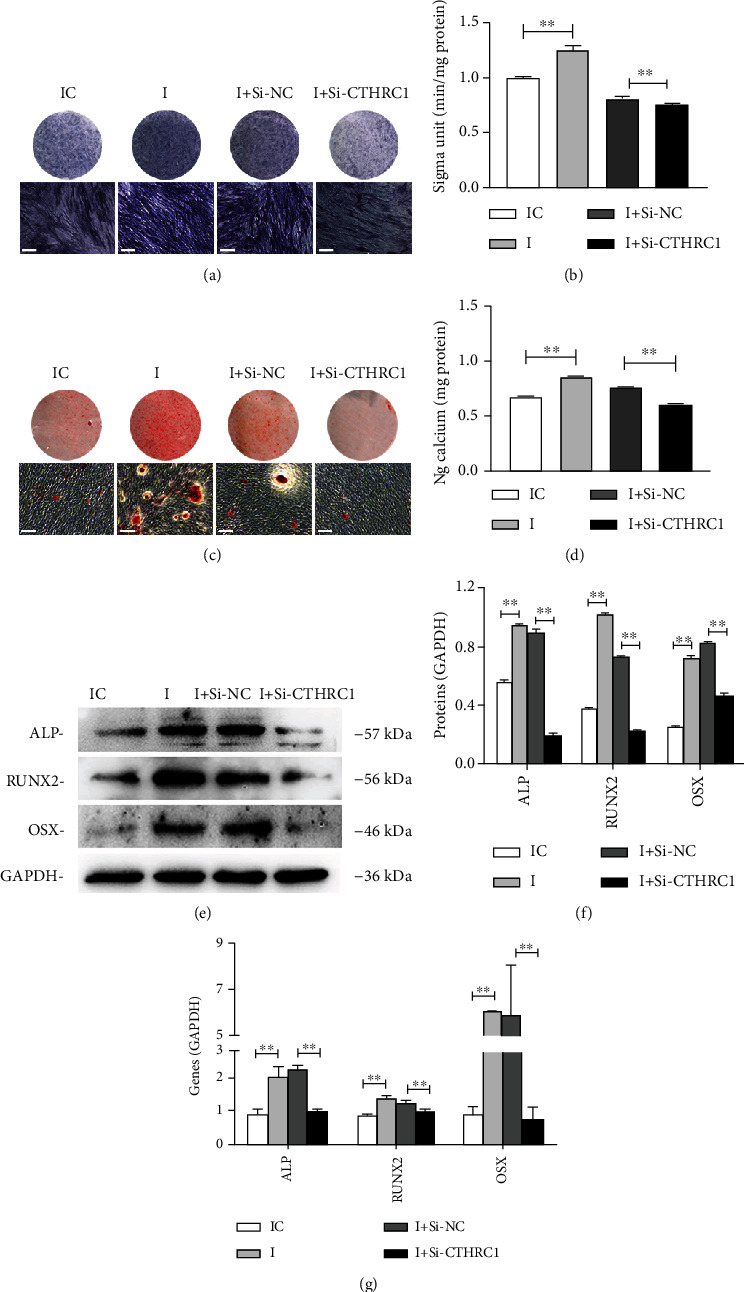
CTHRC1 partially reverses the influence of *hsa-let-7b* on the osteogenic differentiation of PDLSCs. (a, b) ALP staining and ALP activity analysis on day 7 in four group (scale bar = 100 *μ*m). ^∗∗^*P* < 0.01. (c, d) ARS staining and relative CPC assay were performed after osteogenic inducting 14 days (scale bar = 100 *μ*m). ^∗∗^*P* < 0.01. (e) Western blot analysis of ALP, RUNX2, and OSX. (f) Grayscale analyses of (c). ^∗∗^*P* < 0.01. (g) Relative mRNA expression of osteogenic genes. ^∗∗^*P* < 0.01.

To reveal whether *hsa-let-7b* regulated the osteogenic differentiation of PDLSCs by targeting CTHRC1, we cotransfected inhibitor and Si-CTHRC1 into PDLSCs. After osteogenic induction, ALP activity, mineralized nodules, and calcium deposition were increased in group *hsa-let-7b* inhibitor (I) compared with group *hsa-let-7b* inhibitor NC (IC), whereas simultaneously, it was reduced in group *hsa-let-7b* inhibitor+ Si-CTHRC1 (I+ Si-CTHRC1) compared with group *hsa-let-7b* inhibitor+ Si-NC (I+ Si-NC) (*P* < 0.01, Figures 7(a)–7(d)). Similarly, Western blot and qRT-PCR revealed the same results (*P* < 0.01, Figures 7(e)–7(g)).

## 4. Discussion

The ideal treatment of periodontitis is to restore the lost bone tissue, but so far, there is no effective treatment. PDLSCs are a type of stem cells with multidifferentiation potential, which could be considered as ideal candidate cells for bone regeneration applications [5]. Previous studies have shown that miRNAs can regulate the osteogenic differentiation of stem cells. For example, Li et al. [21] showed that miR-144-3p restrains osteogenic differentiation of BMSCs through repression of TET2. Another one conducted by Liu et al. [22] revealed that miR-497-5p upregulates osteogenic differentiation capacity of SCAP. In this study, we first examined the influence of *hsa-let-7b* on osteogenic differentiation in PDLSCs by targeting CTHRC1.

*Let-7* is the first human miRNA to be discovered [10], and it can regulate cell proliferation and differentiation [23]. There have been reports that it plays crucial roles in many biological processes, such as development, differentiation, and cancer [13, 24, 25]. Our results found that *hsa-let-7b* had no significant effect on the proliferation of PDLSCs. But other findings [26] indicated that *let-7b* might inhibit the proliferation of HCC cells, while another research [27] revealed that *let-7b* induced PASMC proliferation. We speculated that the difference in the above results may be related to the difference in cell types. Previous research proved that *hsa-let-7c* affects the differentiation of DPSCs [28]. In this paper, we found that *hsa-let-7b* decreased in PDLSCs after mineralization induction. Subsequently, we changed the expression of *hsa-let-7b* through transfection. On this basis, we investigated whether *hsa-let-7b* affects the osteogenic differentiation of PDLSCs. Results of Western blot and qRT-PCR showed that enhancement of *hsa-let-7b* suppressed the osteogenic differentiation of PDLSCs, which was mainly manifested as a decrease in osteogenic-related markers. However, the inhibition of *hsa-let-7b* promoted osteogenesis. As everyone knows, RUNX2 belongs to the RUNX gene family, which is the key transcriptional regulator for osteogenesis, and directly activates the transcription of osteoblast-related gene (e.g., OSX and ALP) [29, 30]. ALP ang ARS staining further confirmed this trend. This is consistent with our previous research [14].

In order to detect how *hsa-let-7b* regulates the osteogenic differentiation of PDLSCs, we conducted bioinformatics analysis (miRTarBase, miRWalk, miRDB, and TargetScan algorithms). Potential target genes (13,093) of *hsa-let-7b* were predicted by bioinformatic analyses and are related to various biological processes, such as cell differentiation (Figures 4(b)). Unexpectedly, CTHRC1 was the common target gene of *hsa-let-7b* in the 4 databases' prediction. Recent studies have found that CTHRC1 can promote osteogenic differentiation of periodontal ligament stem cells [19]. We have also obtained similar results. Upregulation of CTHRC1 promoted the osteogenic differentiation of PDLSCs, but reducing CTHRC1 took exactly the opposite result. Then, the dual luciferase reporter assay verified that *hsa-let-7b* directly target CTHRC1. Further, *hsa-let-7b* downregulated the expression of CTHRC1 at both mRNA and protein levels in PDLSCs. We also discovered that the osteogenic differentiation of PDLSCs was raised after inhibiting *hsa-let-7b*, while was then reversed after cotransfection with Si-CTHRC1. The above result indicated *hsa-let-7b* depresses the osteogenic differentiation of PDLSCs partially by regulating CTHRC1.

Osteogenic differentiation of MSCs can offer a new therapeutic option for bone tissue engineering [31, 32]. Up to now, the theory of osteogenic differentiation is unclear yet. The process requires the interaction between cytokines, growth factors, and hormones [20]. CTHRC1 has already been proved to stimulate osteoblast differentiation in MSCs [33]. Further studies have proved that CTHRC1 plays its roles through a variety of signaling pathways such as TGF-*β*, PI3K/AKT/ERK, HIF-1*α*, and MAPK signaling pathways [34, 35]. As we all know, TGF-*β* is one of the classics signaling pathways in osteogenesis [32]. According to report, TGF-*β*/BMP2 signaling pathway plays a key role in osteoblast differentiation [36]. Bone morphogenetic protein (BMP) 2 affected osteoblast differentiation by activating the JNK/MAPK pathway [30, 37]. Besides, Zhang et al. has reported that CTHRC1 influences chondrocyte apoptosis via JNK1/2 signaling [38]. In addition, TGF-*β* signaling pathway promotes osteogenesis through cooperation with other signaling pathways (such as MAPK, Smad2/3, and Wnt) [36]. However, the relationship between CTHRC1, TGF-*β* signaling pathway, and osteogenic differentiation in PDLSCs needs deeper study.

In conclusion, inhibition of *hsa-let-7b* in PDLSCs could be considered to promote osteogenic differentiation of PDLSCs. And we proved this effect was achieved by targeting CTHRC1. We hope that this study can provide some assistance in the field of periodontal regeneration. In order to better apply PDLSCs to tissue engineering researches and clinical treatment, much deeper studies are required to detect the mechanism osteogenic differentiation in PDLSCs, or other MSCs.

## Figures and Tables

**Table 1:** The sequences of primer for qRT-PCR analysis.

Target gene	Primes	Sequences (5′-3′)
*ALP*	Forward	GACCTCCTCGGAAGACACTC
	Reverse	TGAAGGGCTTCTTGTCTGTG

*RUNX2*	Forward	TCTTAGAACAAATTCTGCCCTTT
	Reverse	TGCTTTGGTCTTGAAATCACA

*OSX*	Forward	CCTCCTCAGCTCACCTTCTC
	Reverse	GTTGGGAGCCCAAATAGAAA

*CTHRC1*	Forward	TCATCGCACTTCTTCTGTGGA
	Reverse	GCCAACCCAGATAGCAACATC

*GAPDH*	Forward	GAAGGTGAAGGTCGGAGTC
	Reverse	GAGATGGTGATGGGATTTC

## Data Availability

Datasets used and analyzed during the current study are available from the corresponding author on reasonable request.

## References

[1] Liu Y., Liu C. P., Zhang A. K. (2019). Down-regulation of long non-coding RNA MEG3 suppresses osteogenic differentiation of periodontal ligament stem cells (PDLSCs) through miR-27a-3p/IGF1 axis in periodontitis. *Aging*.

[2] Xu Y., Wang Y., Pang X. (2019). Potassium dihydrogen phosphate promotes the proliferation and differentiation of human periodontal ligament stem cells via nuclear factor kappa B pathway. *Experimental Cell Reseach*.

[3] Huang Y., Han Y., Guo R. (2020). Long non-coding RNA FER1L4 promotes osteogenic differentiation of human periodontal ligament stromal cells via miR-874-3p and vascular endothelial growth factor A. *Stem Cell Research & Therapy*.

[4] Yan G. Q., Wang X., Yang F. (2017). MicroRNA-22 promoted osteogenic differentiation of human periodontal ligament stem cells by targeting HDAC6. *Journal of Cellular Biochemistry*.

[5] Li X. B., Zheng Y. F., Zheng Y. (2018). Circular RNA CDR1as regulates osteoblastic differentiation of periodontal ligament stem cells via the miR-7/GDF5/SMAD and p38 MAPK signaling pathway. *Stem Cell Research & Therapy*.

[6] Ding G., Liu Y., Wang W. (2010). Allogeneic periodontal ligament stem cell therapy for periodontitis in swine. *Stem Cells*.

[7] Mrozik K. M., Wada N., Marino V. (2013). Regeneration of periodontal tissues using allogeneic periodontal ligament stem cells in an ovine model. *Regeneration Medicine*.

[8] Chen F. M., Gao L. N., Tian B. M. (2018). Correction to: Treatment of periodontal intrabony defects using autologous periodontal ligament stem cells: a randomized clinical trial. *Stem Cell Research & Therapy*.

[9] Sahin C., Mamillapalli R., Yi K. W., Taylor H. S. (2018). MicroRNA let-7b: a novel treatment for endometriosis. *Journal of Cellular and Molecular Medicine*.

[10] Roush S., Slack F. J. (2008). The let-7 family of microRNAs. *Trends in Cell Biology*.

[11] Huang C., Geng J., Jiang S. (2017). MicroRNAs in regulation of osteogenic differentiation of mesenchymal stem cells. *Cell Tissue Reseach*.

[12] Peter M. E. (2009). Let-7 and miR-200 microRNAs: guardians against pluripotency and cancer progression. *Cell Cycle*.

[13] Lee H., Han S., Kwon C. S., Lee D. (2016). Biogenesis and regulation of the let-7 miRNAs and their functional implications. *Protein & Cell*.

[14] Wang Y., Pang X., Wu J. (2018). MicroRNA hsa-let-7b suppresses the odonto/osteogenic differentiation capacity of stem cells from apical papilla by targeting MMP1. *Journal of Cellular Biochemistry*.

[15] Ding X., Huang R., Zhong Y. (2020). CTHRC1 promotes gastric cancer metastasis via HIF-1*α*/CXCR4 signaling pathway. *Biomedicine & Pharmacotherapy*.

[16] Li J., Wang Y., Ma M. (2019). Autocrine CTHRC1 activates hepatic stellate cells and promotes liver fibrosis by activating TGF-*β* signaling. *eBioMedicine*.

[17] Zhou H. F., Su L. B., Liu C. (2019). CTHRC1 may serve as a prognostic biomarker for hepatocellular carcinoma. *Oncotargets Therapy*.

[18] Stohn J. P., Wang Q. Z., Siviski M. E. (2015). Cthrc1 controls adipose tissue formation, body composition, and physical activity. *Obesity*.

[19] Wang C. Z., Gu W. T., Sun B. Y. (2017). CTHRC1 promotes osteogenic differentiation of periodontal ligament stem cells by regulating TAZ. *Journal of Cellular and Molecular Medicine*.

[20] Park J. W., Fu S. Y., Huang B. R., Xu R. H. (2020). Alternative splicing in mesenchymal stem cell differentiation. *Stem Cells*.

[21] Li N., Liu L., Liu Y., Luo S., Song Y., Fang B. (2020). miR-144-3p suppresses osteogenic differentiation of BMSCs from patients with aplastic anemia through repression of TET2. *Molecular Therapy Nucleic Acids*.

[22] Liu J., Wang X., Song M. (2020). MiR-497-5p regulates osteo/odontogenic differentiation of stem cells from apical papilla via the Smad signaling pathway by targeting Smurf2. *Frontiers in Genetics*.

[23] Bussing I., Slack F. J., Grosshans H. (2008). let-7 microRNAs in development, stem cells and cancer. *Trends in Molecular Medicine*.

[24] Jiang S. (2018). Recent findings regarding let-7 in immunity. *Cancer Letters*.

[25] Chirshev E., Oberg K. C., Ioffe Y. J., Unternaehrer J. J. (2019). Let-7 as biomarker, prognostic indicator, and therapy for precision medicine in cancer. *Clinical and Translational Medicine*.

[26] Hui L., Zheng F., Bo Y. (2020). MicroRNA let-7b inhibits cell proliferation via upregulation of p21 in hepatocellular carcinoma. *Cell & Bioscience*.

[27] Zhang R. F., Su H., Ma X. Q. (2019). MiRNA let-7b promotes the development of hypoxic pulmonary hypertension by targeting ACE2. *American Journal of Physiology Lung Cellular and molecular Physiology*.

[28] Liu G. X., Ma S., Li Y. (2018). Hsa-let-7c controls the committed differentiation of IGF-1-treated mesenchymal stem cells derived from dental pulps by targeting IGF-1R via the MAPK pathways. *Experimental&Molecular Medicine*.

[29] Cohen M. M. (2009). Perspectives onRUNXgenes: an update. *American Journal of Medicne Genetics A*.

[30] Wang Y., Jia Z., Diao S. (2016). IGFBP5 enhances osteogenic differentiation potential of periodontal ligament stem cells and Wharton's jelly umbilical cord stem cells, via the JNK and MEK/Erk signalling pathways. *Cell Proliferation*.

[31] Ju C., Liu R. F., Zhang Y. W. (2019). Mesenchymal stem cell-associated lncRNA in osteogenic differentiation. *Biomedicine & Pharmacotherapy*.

[32] Yang Q. L., Jia L. F., Li X. B. (2018). Long noncoding RNAs: new players in the osteogenic differentiation of bone marrow- and adipose-derived mesenchymal stem cells. *Stem Cell Reviews and Reports*.

[33] Takeshita S., Fumoto T., Matsuoka K. (2013). Osteoclast-secreted CTHRC1 in the coupling of bone resorption to formation. *Journal of Clinical Investigation*.

[34] Mei D., Zhu Y., Zhang L., Wei W. (2020). The role of CTHRC1 in regulation of multiple signaling and tumor progression and metastasis. *Mediators of Inflammation*.

[35] Guo Y., Jiang C., Yao S. (2021). CTHRC1 knockdown promotes inflammatory responses partially by p38 MAPK activation in human periodontal ligament cells. *Inflammation*.

[36] Chen G., Deng C., Li Y. P. (2012). TGF-*β* and BMP signaling in osteoblast differentiation and bone formation. *International Journal of Biological Sciences*.

[37] Guicheux J., Lemonnier J., Ghayor C., Suzuki A., Palmer G., Caverzasio J. (2003). Activation of p38 mitogen-activated protein kinase and c-Jun-NH2-terminal kinase by BMP-2 and their implication in the stimulation of osteoblastic cell differentiation. *Journal of Bone and Mineral Research*.

[38] Zhang Q., Yin Z. S., Zhang F. W., Cao K., Sun H. Y. (2018). CTHRC1 mediates IL‑1*β*‑induced apoptosis in chondrocytes via JNK1/2 signaling. *International Journal of Molecular Medicine*.

